# Molecular Survey of Tick-Borne Pathogens Reveals Diversity and Novel Organisms With Veterinary and Public Health Significance in Wildlife From a National Nature Reserve of China

**DOI:** 10.3389/fvets.2021.682963

**Published:** 2021-07-12

**Authors:** Jifei Yang, Xiaojun Wang, Jinming Wang, Zhijie Liu, Qingli Niu, Muhammad Uzair Mukhtar, Guiquan Guan, Hong Yin

**Affiliations:** ^1^State Key Laboratory of Veterinary Etiological Biology, Key Laboratory of Veterinary Parasitology of Gansu Province, Lanzhou Veterinary Research Institute, Chinese Academy of Agricultural Sciences, Lanzhou, China; ^2^Tangjiahe National Nature Reserve, Qingchuan, China; ^3^Jiangsu Co-innovation Center for Prevention and Control of Important Animal Infectious Diseases and Zoonoses, Yangzhou, China

**Keywords:** tick-borne pathogen, *Theileria*, *Anaplasma bovis*, *Anaplasma phagocytophilum*, wildlife, nature reserve

## Abstract

Wildlife is involved in the maintenance and transmission of various tick-borne pathogens. The objective of the present study was to determine the occurrence and diversity of tick-borne pathogens in free-ranging wild animals collected from Tangjiahe National Nature Reserve of China. Blood or liver samples from 13 wild animals (5 takin, 3 Himalayan goral, 3 Reeves' muntjac, 1 forest musk deer, and 1 wild boar) were collected and screened for piroplasm, *Anaplasma* spp., *Ehrlichia* spp., and spotted fever group (SFG) rickettsiae by PCR-based on different gene loci. Three *Theileria* species, a potential novel *Theileria* parasite (*Theileria* sp. T4) and two *Anaplasma* species were identified in those wildlife. *Theileria capreoli* was found in Himalayan goral, Reeves' muntjac, and forest musk deer; *Theileria luwenshuni, Theileria uilenbergi*, and a potential novel, *Theileria* parasite (*Theileria* sp. T4), were identified in takin. Meanwhile, *Anaplasma bovis* was identified in Himalayan goral, takin, Reeves' muntjac, forest musk deer, and wild boar; *Anaplasma phagocytophilum* and related strains was found in takin, Reeves' muntjac, and forest musk deer. All wildlife included in this study was negative for *Babesia, Anaplasma ovis, Anaplasma marginale, Ehrlichia*, and SFG rickettsiae. Moreover, coinfection involving *Theileria* spp. and *Anaplasma* spp. was observed in eight wild animals. This study provided the first evidence of tick-borne pathogens in free-ranging wild animals from the nature reserve, where contact between domestic and wild animals rarely occurs.

## Introduction

Tick-borne diseases have been becoming increasingly important with the increase in incidences and geographic distribution (1). They cause extensive economic losses in the livestock industry and also impose a growing health burden on human populations worldwide. In nature, the causative agents are maintained in complex transmission cycles involving ticks, various domestic, and wild animals (2). In the past three decades, an increasing number of new species, genotypes, or genetic variants of tick-borne pathogens were identified in ticks and domestic and wild animals, some of which have been subsequently recognized as human pathogens, such as *Anaplasma phagocytophilum*, Candidatus *Neoehrlichia mikurensis, Rickettsia slovaca, Rickettsia raoultii, Rickettsia conorii, Babesia venatorum*, and *Babesia divergens* (3). The global impact of tick-borne diseases on livestock, along with their zoonotic potential, has attracted great attention and interest in the surveillance for ticks and their associated pathogens not only in domestic but also in wild animals.

Wild animals serving as reservoir hosts for various tick-borne infections has been well-established globally. They play a critical role in the maintenance of the endemic cycle of tick-borne diseases, which increase the risk of disease transmission to humans and domestic animals due to their movements potentially affect the pathogens and vectors distribution (3). Therefore, the identification of the wild host species that serve as important reservoirs of tick-borne infections is essential. However, limited information is available on tick-borne pathogens in wild animals when compared with ticks and domestic animals in China and remains to be disclosed, especially in those from nature reserves, where contact between wild and domestic animals rarely occurs. Those animals fulfill an important mission in the persistence of tick vectors and pathogens in nature (4). In the present study, the occurrence and diversity of tick-borne agents with veterinary and medical significance were investigated in wild animals collected from Tangjiahe National Nature Reserve of China, which is a natural habitat for many endangered and national protected wildlife.

## Materials and Methods

### Study Site and Collection of Specimens

The Tangjiahe National Nature Reserve is a protected area located in Qingchuan County of Sichuan Province, southwestern China. The reserve was established in 1978 and dedicated to protecting the habitat and wildlife. Occupying an area of about 40,000 ha, 481 species of vertebrates inhabited in this reserve, including 72 national key protected wildlife. This area is one of the hotspot areas for global biodiversity conservation and is also an important habitat of many endangered wildlife, such as giant pandas, golden monkeys, and takins.

From March 2016 to April 2017, 13 dead wild animals were found in Tangjiahe National Nature Reserve, including takin (*Budorcas taxicolor*, class I national protected species, *n* = 5), forest musk deer (*Moschus berezovskii*, class I national protected species, *n* = 1), Himalayan goral (*Naemorhedus goral*, class II national protected species, *n* = 3), Reeves' muntjac (*Muntiacus reevesi, n* = 3), and wild boar (*Sus scrofa, n* = 1). The ethylenediaminetetraacetic acid (EDTA) blood or liver samples were collected from those animals, and DNA was isolated from 200 μl whole blood or 25 mg liver by using QIAamp DNA Mini Kit according to manufacturer's instructions. The concentration of DNA samples (ng/μl) was measured by using NanoDrop2000 (Thermo Scientific, Waltham, MA, USA).

### PCR Reactions

Polymerase chain reactions were carried out to screen the samples for detection of 18S rRNA sequences of piroplasm; 16S rRNA sequences of *A. phagocytophilum, Anaplasma bovis*, and *Ehrlichia* spp.; *msp4* sequences of *Anaplasma marginale* and *Anaplasma ovis*; and *ompA* sequences of spotted fever group (SFG) rickettsiae, according to previously described (Table 1). The PCR amplifications were performed in an automatic thermocycler (Bio-Rad, Hercules, CA, USA) with a final volume of 25 μl containing 2.5 μl of 10× PCR buffer (Mg^2+^ Plus), 2.0 μl of each deoxyribonucleotide triphosphate (dNTP) at 2.5 mM, 1.0 μl of each primer (10 pmol), 2.0 μl of DNA samples (blood DNA, 18.4–24.3 ng/μl; liver DNA, 94.8–134.2 ng/μl), 1.25 U of Taq DNA polymerase (TaKaRa, Dalian, China), and 16.25 μl of distilled water. Positive (containing DNA of the corresponding organisms) and negative controls were included in all amplifications. PCR products were analyzed by agarose gel electrophoresis (1.2%) and visualized under UV transillumination after Goldview staining (Solarbio, Beijing, China).

**Table 1:** Primers and PCR amplification conditions.

**Pathogen**	**Target gene**	**Primer name**	**Primer Sequence (5′-3′)**	**Annealing temp (°C)**	**Amplicon size (bp)**	**References**
Piroplasm	18S rRNA	Piro1-S	CTTGACGGTAGGGTATTGGC	55	~1,410	(5, 6)
		Piro3-AS	CCTTCCTTTAAGTGATAAGGTTCAC			
	18S rRNA	PIRO-A1	CGCAAATTACCCAATCCTGACA	55	~430	
		PIRO-B	TTAAATACGAATGCCCCCAAC			
*A. phagocytophilum*	16S rRNA	EE1	CCTGGCTCAGAACGAACGCTGGCGGC	55	1,433	(7, 8)
		EE2	AGTCACTGACCCAACCTTAAATGGCTG			
	16S rRNA	SSAP2f	GCTGAATGTGGGGATAATTTAT	60	641	
		SSAP2r	ATGGCTGCTTCCTTTCGGTTA			
*A. bovis*	16S rRNA	EE1	TCCTGGCTCAGAACGAACGCTGGCGGC	55	1,433	(7, 8)
		EE2	AGTCACTGACCCAACCTTAAATGGCTG			
	16S rRNA	AB1f	CTCGTAGCTTGCTATGAGAAC	60	551	
		AB1r	TCTCCCGGACTCCAGTCTG			
*A. marginale*	*msp4*	AmargMSP4Fw	CTGAAGGGGGAGTAATGGG	60	344	(9)
		AmargMSP4Rev	GGTAATAGCTGCCAGAGATTCC			
*A. ovis*	*msp4*	MSP45	GGGAGCTCCTATGAATTACAGAGAATTGTTTAC	55	869	(10)
		MSP43	CCGGATCCTTAGCTGAACAGAATCTTGC			
*Ehrlichia* spp.	16S rRNA	ECC	AGAACGAACGCTGGCGGCAAGC	60	450	(7)
		ECB	CGTATTACCGCGGCTGCTGGCA			
SFG rickettsiae	*OmpA*	Rr190.70	ATGGCGAATATTTCTCCAAAA	55	632	(11)
		Rr190.701	GTTCCGTTAATGGCAGCATCT			

### DNA Sequencing and Phylogenetic Analysis

The positive amplicons were purified from agarose gel with the AxyPrepTM DNA Gel Extraction Kit (Axygen, Union City, CA, USA) according to the manufacturer's instructions. Purified products were cloned into pGEM-T Easy vector (Promega, Madison, WI, USA) and transformed into *Escherichia coli JM109* competent cells (TaKaRa). Three clones were selected randomly for sequencing (GenScript, Nanjing, China). The obtained sequences were compared with those deposited in GenBank using BLASTn, and multiple-sequence alignments were constructed by using ClustalW algorithm in the MegAlign program of the Lasergene software (DNASTAR, Madison, WI, USA). Phylogenetic relationships were assessed by using neighbor-joining (NJ) method with the Kimura two-parameter model, and the bootstrap test was replicated 1,000 times (12).

### Compliance With Ethical Standards

Animal treatments and sample preparation complied with the Animal Ethics Procedures and Guidelines and was approved by the Animal Ethics Committee of Lanzhou Veterinary Research Institute, Chinese Academy of Agricultural Sciences.

### Nucleotide Sequence Accession Numbers

The nucleotide sequences obtained in this study were deposited in the GenBank database and designated accession numbers: MH179333 for 18S rRNA gene sequence of *Theileria uilenbergi*, MH179334 and MH179335 for 18S rRNA gene sequences of *Theileria capreoli*, MH179336 for 18S rRNA gene sequence of *Theileria luwenshuni*, MH179337 for 18S rRNA gene sequence of *Theileria* sp. T4; MH180816 and MH180817 for 16S rRNA gene sequences of *A. bovis*, and MH180818–MH180820 for 16S rRNA gene sequences of *A*. *phagocytophilum*.

## Results

In the present study, piroplasms, *A. bovis* and *A. phagocytophilum*, were detected in 9, 10, and 4 of 13 wildlife included in this study (Table 2). The partial 18S rRNA gene sequences (~430 bp) of piroplasm (*Babesia*/*Theileria*) were amplified, and sequence analysis revealed three *Theileria* species and a potential novel *Theileria* parasite in those wildlife. No *Babesia* sequence was found in those amplicons. *Theileria capreoli* was found in one Himalayan goral, two Reeves' muntjac, and one forest musk deer (Table 2). The 18S rRNA sequences of *T. capreoli* from Himalayan goral showed 98.7% identity with *T. capreoli* isolate Qilian-Mountain 13 (KJ188219) (13); the isolates from Reeves' muntjac and forest musk deer showed 100% identity with *T. capreoli* isolate 8P (KJ451469) (5). *Theileria luwenshuni* was identified in three takins, and all three isolates were 100% identical to the *T. luwenshuni* isolate Dawu B5 (JX469514) (14). *Theileria uilenbergi* (P6-a, MH179333) was found in one takin; it was 99.7% identical to the *T. uilenbergi* isolate Li 2 (JF719835). A potential novel *Theileria* parasite (*Theileria* sp. T4) identified in one takin showed 99.7% identity with the *Theileria* sp. Iwate 2-2 (AB602881).

**Table 2:** The piroplasm and *Anaplasma* spp. identified in wildlife from Tangjiahe National Nature Reserve, China.

**Animal species**	**Scientific name**	**Sample**	**Piroplasm**	***A. bovis***	***A. phagocytophilum***
Himalayan goral	*Naemorhedus goral*	Blood	*T. capreoli*	+	–
Himalayan goral	*Naemorhedus goral*	Liver	–	–	–
Himalayan goral	*Naemorhedus goral*	Liver	–	–	–
Takin	*Budorcas taxicolor*	Blood	*Theileria* sp. T4	+	+
Takin	*Budorcas taxicolor*	Blood	*T. uilenbergi*	+	+
Takin	*Budorcas taxicolor*	Blood	*T. luwenshuni*	+	–
Takin	*Budorcas taxicolor*	Liver	*T. luwenshuni*	+	–
Takin	*Budorcas taxicolor*	Liver	*T. luwenshuni*	–	–
Reeves' muntjac	*Muntiacus reevesi*	Blood	–	+	+
Reeves' muntjac	*Muntiacus reevesi*	Blood	*T. capreoli*	+	–
Reeves' muntjac	*Muntiacus reevesi*	Liver	*T. capreoli*	+	–
Forest musk deer	*Moschus berezovskii*	Blood	*T. capreoli*	+	+
Wild boar	*Sus scrofa*	Liver	–	+	–

**Figure 1:**
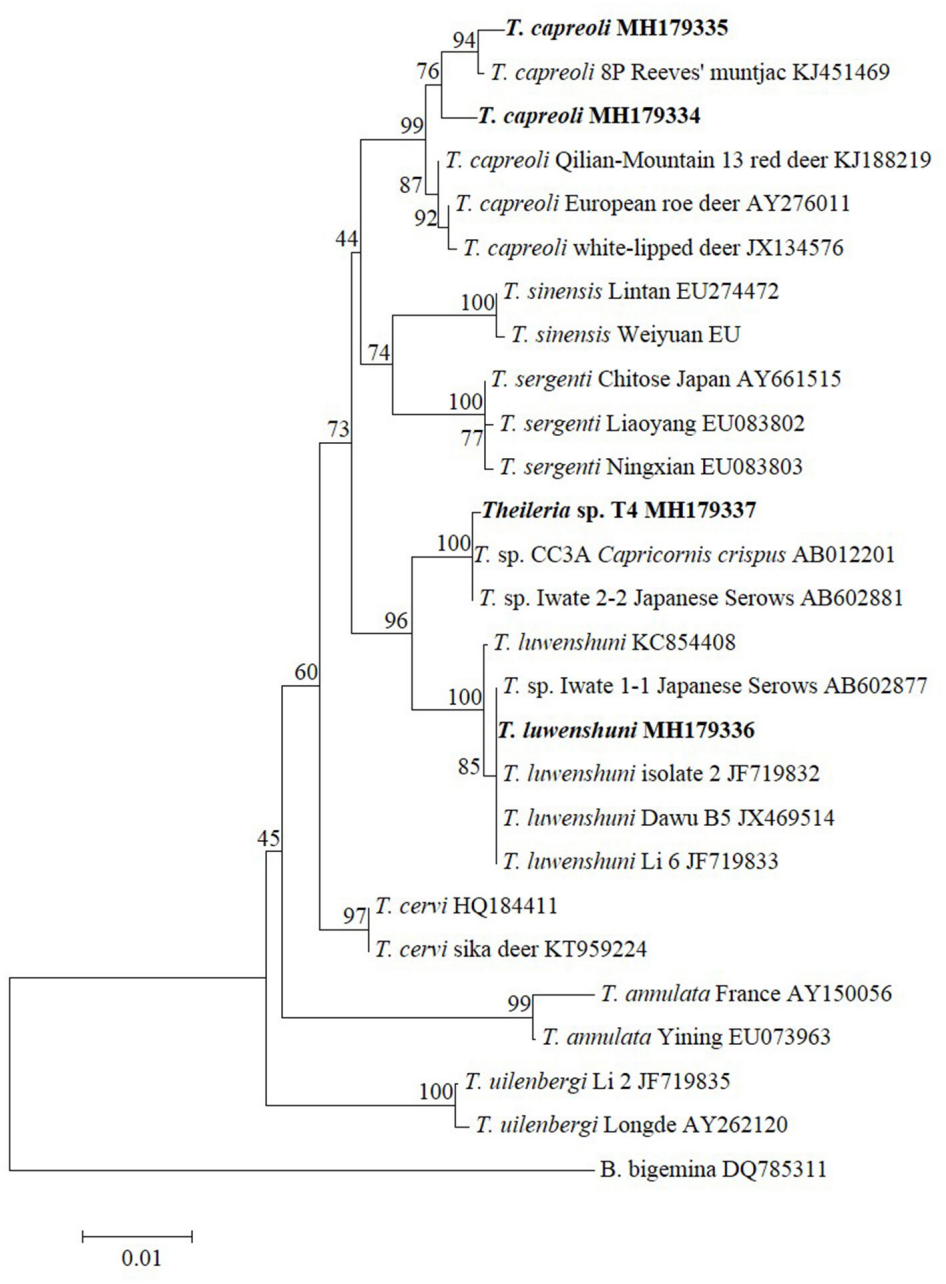
Phylogenetic analysis of the *Theileria* species identified in this study based on the 18S rRNA gene. *Babesia bigemina* was used as outgroup. Boldface indicate the sequences obtained in this study.

To further verify the *Theileria* species identified in this study, ~1,410-bp fragments for 18S RNA of *T. capreoli, T. luwenshuni*, and *Theileria* sp. T4 were amplified, whereas no amplicons were detected for *T. uilenbergi* (P6-a, MH179333). Sequence and phylogenetic analysis revealed that the *T. capreoli* isolates (Pb2-a, MH179334; Pb11-a, MH179335) were clustered together with the isolates from Reeves' muntjac (KJ451469), red deer (KJ188219), European roe deer (AY276011), and white-lipped deer (JX134576) with 99.0–99.7% sequence identity; *Theileria* sp. T4 (MH179337) was closely related to the strains CC3A (AB012201) and Iwate (AB602881) of an unclassified *Theileria* species detected from *Capricornis crispus* and Japanese serows with 99.9% sequence identity, respectively; *T. luwenshuni* (Pb7-a, MH179336) was closely related to the *T. luwenshuni* isolate 2 (JF719832), Dawu B5 (JX469514), Li 6 (JF719833), and Iwate 1-1 (AB602877) with 100% sequence identity (Figure 1).

*Anaplasma bovis* was detected in one Himalayan goral, four takin, three Reeves' muntjac, one forest musk deer, and one wild boar (Table 2). The 16S rRNA sequences of *A. bovis* obtained from positive samples were 99.6–100% identical to each other. Three isolates (Ab4a, MH180817) from two takins and one forest musk deer were 100% identical to the *A. bovis* isolates KWDAB2 (GU556627), 2-44Ab (KF465981), AB-KGHL (AF470698), C_AP_1-2 (LC068731), and ES1 (KC811530) derived from Korean water deer, Reeves' muntjac, *Haemaphysalis longicornis*, sika deer, and *Elephantulus myurus*, respectively. The remaining isolates (Ab2a, MH180816) were 100% identical to *A. bovis* isolates b1 (KX115423), b2-25a (MF066914), Ab3a (KJ639883), NX1-1 (KM186945), T-KOAB1 (KC311344), giraffe 2013-5 (KU870666), and JC4-4 (KM186944) from cattle, sheep, red deer, roe deer, *H. longicornis*, giraffe, and *Procapra gutturosa*, respectively.

*Anaplasma phagocytophilum* infection was found in two takins, one Reeves' muntjac, and one forest musk deer (Table 2). Three 16S RNA sequence variants of *A. phagocytophilum* were identified that shared 98.5–99.8% sequence similarity. The 16S RNA sequence of variant 1 (Ap4a, MH180818) and variant 2 (Ap5-b, MH180819) detected from takin showed 99.8–100% identity with *A. phagocytophilum*-like 1 strain derived from cattle (KX702974) from Tunisia (15). The variant 3 (Ap11-b, MH180820) identified from Reeves' muntjac and forest musk deer was 100% identical to the *A. phagocytophilum* isolate detected from white yaks (KT824826).

All wildlife included in this study were negative for *A. ovis, A. marginale, Ehrlichia* spp., and SFG rickettsiae. The coinfection of *Theileria* spp. and *Anaplasma* spp. was found in eight wildlife, including one Himalayan goral, four takins, two Reeves' muntjac, and one forest musk deer; the coinfection of two *Anaplasma* species (*A. bovis* and *A. phagocytophilum*) was observed in two takins, one Reeves' muntjac, and one forest musk deer (Table 2).

## Discussion

Wild animals carry various tick species and act as important reservoir hosts for many tick-borne pathogens. The present study investigated tick-borne bacteria and protozoan parasites, and three *Theileria* (*T. capreoli, T. uilenbergi*, and *T. luwenshuni*), a potential novel *Theileria* parasite (*Theileria* sp. T4), and two *Anaplasma* species (*A. bovis, A. phagocytophilum*, and related strains) were identified in wildlife from Tangjiahe National Nature Reserve situated in Sichuan Province, southwestern China.

*Theileria* is tick-borne protozoa, infecting both leukocytes and erythrocytes of their vertebrate hosts (16). Domestic and wild animals are susceptible to different *Theileria* species, many of which are previously known to be reasonably host specific. However, increasing evidence showed that some *Theileria* species have been identified in unexpected hosts from different geographic locations, especially in wild animals. In this study, *T. capreoli* was identified in Himalayan goral, Reeves' muntjac, and forest musk deer. This organism is initially described from roe deer and is considered to be a benign parasite of wild cervids (17); the infections have also been recorded in fallow deer, red deer, and Chinese water deer (17, 18). *Theileria uilenbergi* and *T. luwenshuni* are considered to be highly pathogenic parasites of small domestic ruminants (19). The occurrence of these two *Theileria* species has been widely reported in sheep and goats from China and causes great economic losses for the livestock industry (19). However, there are sporadic reports of *T. uilenbergi* and *T. luwenshuni* in wild animals. To date, infection of *T. luwenshuni* (*Theileria* sp. China 1) was observed in a few Chinese water deer and roe deer from South Korea, roe deer, sika deer, and red deer from China (18, 20, 21), while *T. uilenbergi* has been detected in red deer, sika deer, and Reeves' muntjac from China (5, 13). The present study constitutes the first evidence of *T. luwenshuni* and *T. uilenbergi* infections in takins. As mentioned above, these two *Theileria* parasites are high pathogenic for sheep and goats; however, their pathogenicity for wild animals is still unclear, which warrants further investigation. Moreover, a potential novel *Theileria* parasite (*Theileria* sp. T4) was identified in takin from this study that has been previously reported in *Capricornis crispus* (AB012201) and Japanese serows (AB602881).

The members in the genus *Anaplasma* are obligate intracellular bacteria that replicate in various cell types (22). These agents are associated with infections in humans and many species of domestic and wild animals. Of those well-known *Anaplasma* species, *A. bovis* and *A. phagocytophilum* infect a wide range of both domestic and wild animals, whereas the remaining *Anaplasma* species are relatively host specific (23). Since *A. bovis* was first described in cattle, the infection has also been recognized in other ruminant and non-ruminant animals, such as sheep, goats, various deer, Mongolian gazelle, wild boars, dogs, raccoons, and cotton-tail rabbits, (24–26). In the present study, *A. bovis* was detected in all five wild animal species investigated from Tangjiahe National Nature Reserve. These results indicated that Himalayan goral, takin, Reeves' muntjac, forest musk deer, and wild boar can act as reservoir hosts for *A. bovis*.

*Anaplasma phagocytophilum* is a well-known intragranulocytic bacteria with medical and veterinary importance worldwide. The host range of this organism is wide, including rodents, ruminants, carnivores, birds, and humans (23). Currently, there is no evidence for transovarial transmission of *A. phagocytophilum* in tick vectors, suggesting the critical role of reservoir hosts in the maintenance and transmission of the pathogen. To date, although the occurrence of *A. phagocytophilum* has been widely reported in domestic animals in China (27), limited information was available in wild animals, especially wild ruminants. Several species of wild ruminants have been recognized as competent hosts for *A. phagocytophilum*, such as white-tailed deer in the US and roe deer and red deer in Europe (28). In the present study, *A. phagocytophilum* was identified in takin, Reeves' muntjac, and forest musk deer, indicating that these animals can act as a source of infections and dispersal of the agent. Previous reports have demonstrated that *A. phagocytophilum* could be subdivided into genetic variants based on different gene loci, which have different host tropisms and may be involved in different enzootic cycles (23). In the present study, three 16S rRNA variants of *A. phagocytophilum* were obtained from those wild animals. The variants 1 and 2 identified from takin were closely related to the *A. phagocytophilum*-like 1 strains that have been previously found in cattle, sheep, and goats (15, 29). It has been suggested that *A. phagocytophilum*-like 1 strains are considered non-pathogenic for ruminants (15, 29). The variant 3 identified from Reeves' muntjac and forest musk deer was only documented in white yaks (30).

Our results first reported tick-borne pathogens in wild vertebrates that inhabit in a conservation area in southwest China. The free-ranging wild animals infected with *Theileria* and *Anaplasma* species identified herein may act as reservoir hosts for the subsequent spread of those agents. These findings provide relevant information for wildlife conservation and are also important for evaluating the risk of disease transmission between wild animals and livestock/humans.

## Data Availability Statement

The datasets presented in this study can be found in online repositories. The names of the repository/repositories and accession number(s) can be found below: https://www.ncbi.nlm.nih.gov/genbank/, MH179333; https://www.ncbi.nlm.nih.gov/genbank/, MH179334; https://www.ncbi.nlm.nih.gov/genbank/, MH179335; https://www.ncbi.nlm.nih.gov/genbank/, MH179336; https://www.ncbi.nlm.nih.gov/genbank/, MH179337; https://www.ncbi.nlm.nih.gov/genbank/, MH180816; https://www.ncbi.nlm.nih.gov/genbank/, MH180817; https://www.ncbi.nlm.nih.gov/genbank/, MH180818; https://www.ncbi.nlm.nih.gov/genbank/, MH180819; https://www.ncbi.nlm.nih.gov/genbank/, MH180820.

## Ethics Statement

The animal study was reviewed and approved by Animal Ethics Committee of Lanzhou Veterinary Research Institute, Chinese Academy of Agricultural Sciences.

## Author Contributions

HY and JY designed this study and critically revised the manuscript. JY, XW, QN, and MM participated in sample collection and DNA preparation. JY, JW, QN, and GG performed the experiments, data analysis, and drafted the manuscript. ZL and GG participated in the coordination and manuscript revision. All authors read and approved the final manuscript.

## Conflict of Interest

The authors declare that the research was conducted in the absence of any commercial or financial relationships that could be construed as a potential conflict of interest.
